# Eye movements and brain oscillations to symbolic safety signs with different comprehensibility

**DOI:** 10.1186/s40101-015-0081-3

**Published:** 2015-12-10

**Authors:** Yohana Siswandari, Shuping Xiong

**Affiliations:** Department of Human and Systems Engineering, School of Design and Human Engineering, Ulsan National Institute of Science and Technology (UNIST), Ulsan, 689-798 South Korea; Department of Industrial and Systems Engineering, College of Engineering, Korea Advanced Institute of Science and Technology (KAIST), Daejeon, 34141 South Korea

**Keywords:** Safety sign, Comprehensibility, Eye movement, Brain activity, Physiological measures

## Abstract

**Background:**

The aim of this study was to investigate eye movements and brain oscillations to symbolic safety signs with different comprehensibility.

**Methods:**

Forty-two young adults participated in this study, and ten traffic symbols consisting of easy-to-comprehend and hard-to-comprehend signs were used as stimuli. During the sign comprehension test, real-time eye movements and spontaneous brain activity [electroencephalogram (EEG) data] were simultaneously recorded.

**Results:**

The comprehensibility level of symbolic traffic signs significantly affects eye movements and EEG spectral power. The harder to comprehend the sign is, the slower the blink rate, the larger the pupil diameter, and the longer the time to first fixation. Noticeable differences on EEG spectral power between easy-to-comprehend and hard-to-comprehend signs are observed in the prefrontal and visual cortex of the human brain.

**Conclusions:**

Sign comprehensibility has significant effects on real-time nonintrusive eye movements and brain oscillations. These findings demonstrate the potential to integrate physiological measures from eye movements and brain oscillations with existing evaluation methods in assessing the comprehensibility of symbolic safety signs.

## Background

**Fig. 1:**
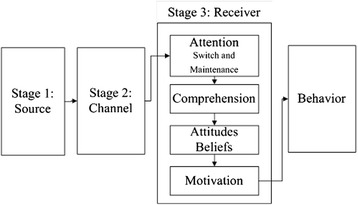
Communications–human information processing (C-HIP) model introduced by Wogalter et al. [1]

Safety signs have been widely used to deliver warning messages to their intended users, in order to prevent dangerous situations. The safety sign comprehension process itself can be regarded as a part of the “communications–human information processing” or C-HIP model (Fig. 1) established by Wogalter et al. [1]. This model is a framework which describes the stages involved as information flows from a source to a receiver, who will process the information, and subsequently produces behavior. Basing on a communication theory, this model developed three conceptual stages: *source*, *channel*, and *receiver*. Each stage of the C-HIP model allows information to be carried out to the next stage, or it can produce a “bottleneck” which will block the flow that will affect the end of the process, which is behavioral compliance. In order to get the “receiver” to react accordingly to a specific message or information, the whole procedure should be completed in a correct manner. A “bottleneck” or misunderstanding that occurs during one or more stages in this C-HIP model would result in the original message or information being perceived wrongly, which will lead to unintended behavior of the information receiver. The first stage, *source*, is the origin or initial transmitter of the risk information, which can be a person(s) or an organized entity (e.g., government). The second stage, *channel*, consists of two basic dimensions. One concerns the media in which the information is shown (e.g., posters, brochures, and labels). Whereas the second dimension concerns the sensory modality used by the receiver to capture this information. The *receiver* stage is further broken down into substages: attention switch and maintenance, comprehension, beliefs and attitudes, and motivation to carry out the compliance behavior (Fig. 1).

Out of all the substages in *receive*r stage, comprehension can be regarded as the most substantial since the correct information flow from this substage plays a big role in transmitting the intended information to the receiver. The faulty design of safety signs could lead to increased potential risks or hazards in public places. For example, a study by Gazmarian et al. [2] with 2659 hospital patients showed that 54 % patients with low health literacy could not understand instructions to take medication on an empty stomach, and 48 % did not understand to take medication every 6 h. Another study by Kirmizioglu and Tuydes-Yaman [3] reported that one major factor affecting safe driving is the comprehensibility of traffic signs by drivers. The results of these studies have affirmed the fact that safety sign comprehension is of utmost importance. Therefore, comprehensibility of safety signs should be ensured prior to being implemented in various public places.

Various evaluation methods have been developed in the past to measure the comprehensibility of the safety sign [4–10]; most of them rely on the guessability score and sometimes cognitive sign features. ISO 9186-1 specifies a method for testing the comprehensibility of graphical symbols and uses the guessability score (GS) from open-ended responses as a measure of the degree to which a graphical symbol communicates its intended message to recipients [8]. In ISO 9186-1, each open-ended response needs be assigned to fully correct, partially correct, or wrong to assess the comprehensibility in terms of GS, as a standard in pictorial comprehension test. However, there is unavoidable subjectivity of the scoring method for evaluating the participants’ open-ended responses in the process of obtaining GS, especially for “partially correct” answers [11]. More importantly, this evaluation method involves questions which require the users to give their opinions or ratings on the tested stimuli after they have “experienced” the stimuli. However, these opinions may be distorted by memory and consciousness effects [12].

Previous studies showed that changes in various physiological processes and states covary with changes in cognitive load elicited by stimuli or objects of attention [13, 14]. There are several advantages of utilizing physiological measures to infer cognitive load: (1) these measures are relatively unobtrusive, (2) these measures do not require overt performance, and (3) most of physiological measures are continuously recorded; they provide us the chance to present measures that respond relatively quickly to phasic shifts in cognitive process [15]. Relating to the aforementioned C-HIP model, some physiological measures seem to correspond with certain stages. Eye tracking data and measures were investigated in this study since people use their visual modality (*stage 2*: *channel*) to capture information conveyed by safety signs. Moreover, eye tracking has been used as an effective tool in a wide variety of studies since it serves as a “mirror” to understanding human attention and behavior when engaged in a specific activity [16, 17]. Brain activity was also investigated since it is closely linked with *comprehension* in the *receiver* stage and it provides a “window” into the human mind [17–21].

The primary purpose of the current study was to investigate eye movements and brain oscillations to symbolic safety signs with different comprehensibility. The findings of this study could provide preliminary evidences on integrating potential physiological measures from eye movements and brain oscillations with existing evaluation methods in assessing the comprehensibility of the symbolic safety signs.

## Methods

### Participants

Forty-two young Korean adults (24 males: 21.2 ± 1.44 years old; 18 females: 20.2 ± 1.31 years old) participated in this study. Each participant provided written informed consent on a protocol approved by the university institutional review board (IRB No.14-17-01-A). The selection criteria are that they should have normal or corrected-to-normal vision, have no colorblindness and red-green deficiencies, and are right-handed. Additionally, participants were instructed not to consume any drinks containing alcohol or caffeine 24 h before their scheduled experiment time, to decrease the likelihood of participants being intoxicated during the experiment.

### Experimental stimuli

**Fig. 2:**
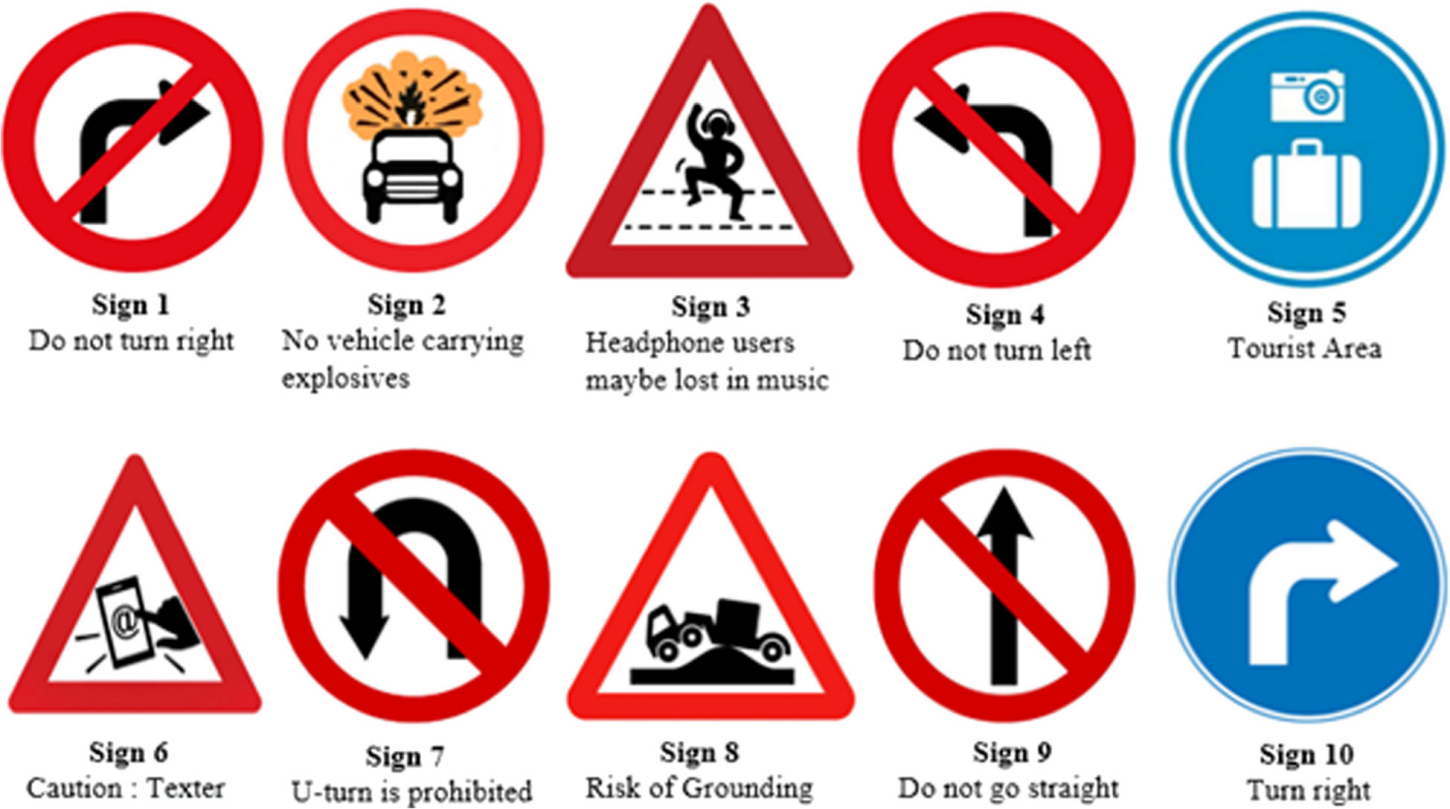
Ten symbolic traffic signs and their intended meanings (source: Know your traffic signs, Department of Transport, UK. www.gov.uk/)

Ten symbolic traffic signs (Fig. 2) were used as stimuli in this study. Among those signs, five widely used road signs (S1—do not turn right; S4—do not turn left; S7—U-turn is prohibited; S9—do not go straight; S10—turn right) were hypothesized to be easy to comprehend, and the other five new road signs in UK (S2—no vehicle carrying explosives; S3—headphone users may be lost in music; S5—tourist area; S6—caution texter; S8—risk of grounding) were hypothesized to be hard to comprehend.

### Experimental procedure

**Fig. 3:**
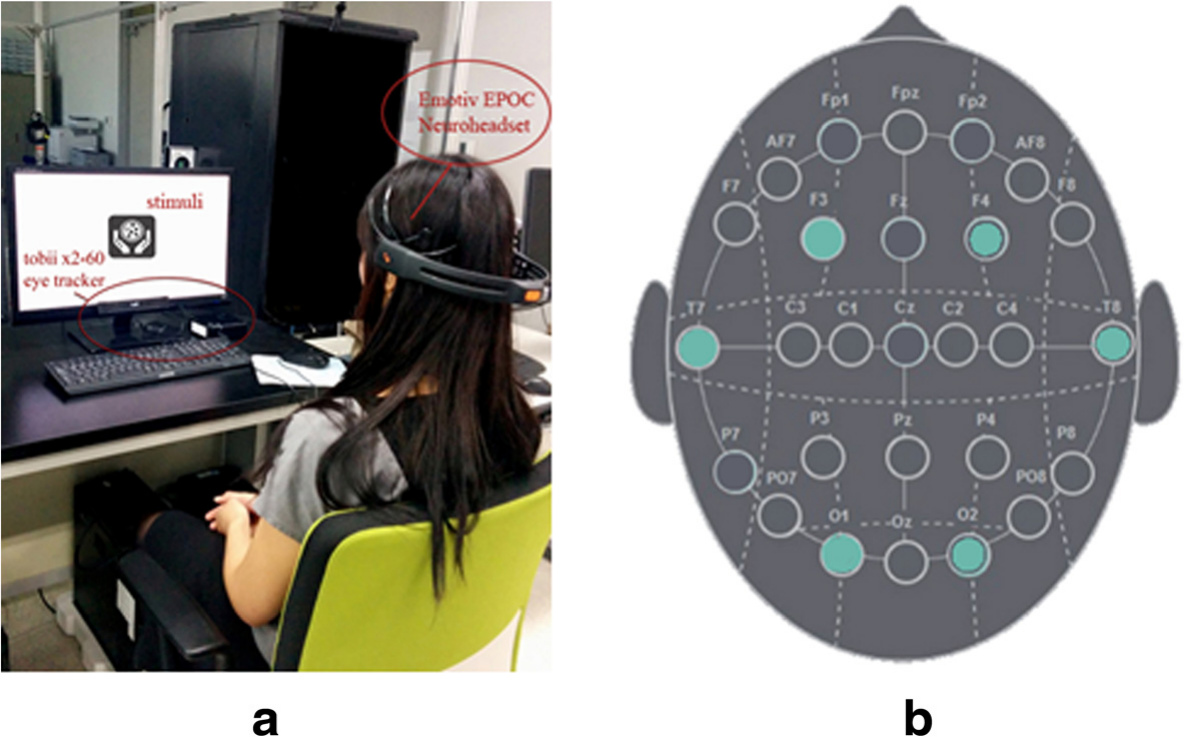
Experimental setup for recording eye movement and brain activity data during the comprehension test (**a**) and six investigated channels shown in *solid circles* (**b**)

After a practice session to familiarize the participant with the setup and experimental procedure, comprehension tests were conducted for ten traffic symbols and the order of the displayed symbols was randomized. During the comprehension test, investigated signs were displayed using Tobii studio software (Tobii Technology), and the participant was asked to guess its actual meaning in an open-ended test [10, 22, 23]. Each participant was given a chance to look at the sign for 10 s, and after that, a new screen would prompt the question “What does the sign mean?” and participants had to give their answers verbally in Korean and ended it with “kkeut” (a Korean term for “ended”). During the 10-s period of sign comprehension, each participant’s real-time physiological data of eye movements and brain activity were simultaneously captured with Tobii X2-60 eye tracker and Emotiv EPOC neuroheadset, respectively (Fig. 3a). The internationally standardized 10-20 system is employed to record the spontaneous electroencephalogram (EEG), prioritizing on six channels (Fig. 3b) which were hypothesized to be linked with cognitive load (F3, F4), visual stimuli processing (O1, O2), and auditory stimuli processing (T7, T8; used as the baseline). For best performance, distance from the participant’s eyes should be approximately 60–65 cm, and his/her gaze angle should not exceed 36° when the participant was located around 65 cm from the eye tracker. To comply with the angle requirement, an adjustable chair was used for the participant. All participants were told not to make unnecessary body movements and not to look away from the screen during the 10-s viewing time, in order to ensure the quality of physiological data recording.

The EEG and eye tracker started to record each participant’s data right after stimuli onset, and stopped after 10 s had ended for each sign. No physiological data was recorded when participants were giving their answers verbally. This procedure was repeated for ten signs. The whole session was recorded by a video camera and also an android-based voice-recorder app, Easy Voice Recorder Pro. The whole experiment lasted for approximately 1 h.

### Data processing and statistical analysis

To calculate the guessability score of each sign, a scoring procedure was carried out as follows. Two judges independently assessed the accuracy of the responses given by the participants. Correct understanding of the actual meaning of the sign symbol (over 80 % understood) was given one point, getting very close to the actual meaning (understood 66–80 %) was given 0.75 points, and getting close to the actual meaning (understood 50–65 %) was given 0.5 points. Giving the opposite answer to the actual meaning caused a one-point deduction, and zero points were awarded for any other answer [24]. Analysis of variance (ANOVA) was conducted to see whether there was significant difference in the comprehensibility among signs. A Bonferroni post hoc test was conducted to group the signs based on their comprehensibility. Signs with a guessability score of at least 85 % were regarded as easy-to-comprehend signs, ie., good signs (ANSI Z535.3 [25]), and signs with a guessability score below 40 % were regarded as hard-to-comprehend signs (bad signs).

Three eye-movement measurements from eye tracking data were investigated: blink rate, pupil diameter, and time to first fixation. For each measurement of a particular participant, the result was averaged across different signs within the same comprehension group (good or bad signs). Pupil diameters for good and bad signs were investigated for 0.5–1s time interval after stimulus onset. The 0.5–1 s was chosen as the observed time interval since the pupil can react to stimuli in 0.2 s, with the response peaking in 0.5–1 s after stimuli onset [26, 27].

Power spectral analysis was conducted to analyze EEG data [18]. First, raw data from six investigated channels for each sign was extracted using an interactive Matlab toolbox for EEG signal processing (EEGLAB). Fast Fourier transform (FFT) was then applied to transform the raw data to a frequency spectrum, resulting in frequency (Hz) on the *x*-axis and power (μV^2^/s) on the *y*-axis. After that, the FFT-ed data was divided into different bands according to the natural frequencies of the brain: delta (0.5–3 Hz), theta (3.5–7 Hz), alpha (8–13 Hz), and gamma (30–70 Hz) [28]. For each participant, the average power of each frequency band across signs which belong to the same comprehension group was calculated for each channel. Therefore, the EEG data of each participant for further statistical analysis was the averaged delta, theta, alpha, and gamma signal power for the two groups of signs with different comprehensibility, for each investigated channel. To examine the hemispheric lateralization during sign comprehension, the right (*R*) hemisphere vs. left (*L*) hemisphere power asymmetry indices for each frequency band were computed for three homologous sites (F4–F3, O2–O1, T8–T7), using the formula of (*R* − *L*)/(*R* + *L*) [29]. Laterality itself was described as qualitatively different functions from homologues areas in the left and right hemispheres [30].

Paired *t* tests were conducted to check whether different comprehensibility levels (easy-to-comprehend and hard-to-comprehend) of the tested signs affect eye tracking and EEG measures. An additional one-sample *t* test was performed on inter-hemispheric power asymmetry indices to check the significance of the hemispheric lateralization. SAS JMP 10 was used for statistical analyses at a significance level of 0.05.

## Results

### Guessability score

The descriptive statistics of guessability scores for ten traffic signs are shown in Table 1. ANOVA results showed that the guessability score differed significantly among different signs [F(9,41) = 321.09, *p* < 0.001], and Bonferroni post hoc grouping analysis showed two different groups (A for easy-to-comprehend signs; B for hard-to-comprehend signs) in terms of comprehensibility. Five signs (signs 1, 4, 7, 9, 10) were classified as easy-to-comprehend signs (good signs), while the other five signs (signs 2, 3, 5, 6, 8) were classified as hard-to-comprehend signs (bad signs). The guessability scores for good signs were all higher than 97 % (higher than the recommended 85 % score by ANSI Z535.3), and the guessability scores for bad signs were all less than 12 %, which showed big differences in comprehensibility between these two groups of signs.

**Table 1:** Descriptive statistics of guessability scores for ten symbolic traffic signs

Sign no.	Intended meaning	Guessability score (mean ± SD) (%)	Bonferroni grouping result^a^
4	Do not turn left	100 ± 0.0	A (easy-to-comprehend group—good signs)
10	Turn right	100 ± 0.0	
1	Do not turn right	99.4 ± 3.9	
9	Do not go straight	98.2 ± 8.5	
7	U turn is prohibited	97.6 ± 15.4	
5	Tourist area	11.3 ± 25.4	B (hard-to-comprehend group—bad signs)
2	No vehicle carrying explosives	6.8 ± 39.2	
3	Headphone users may be lost in music	6.3 ± 15.5	
8	Risk of grounding	2.9 ± 9.9	
6	Caution: texter	2.4 ± 15.6	

^a^Different letters indicate significant group differences

### Eye tracking measures

#### Blink rate

**Fig. 4:**
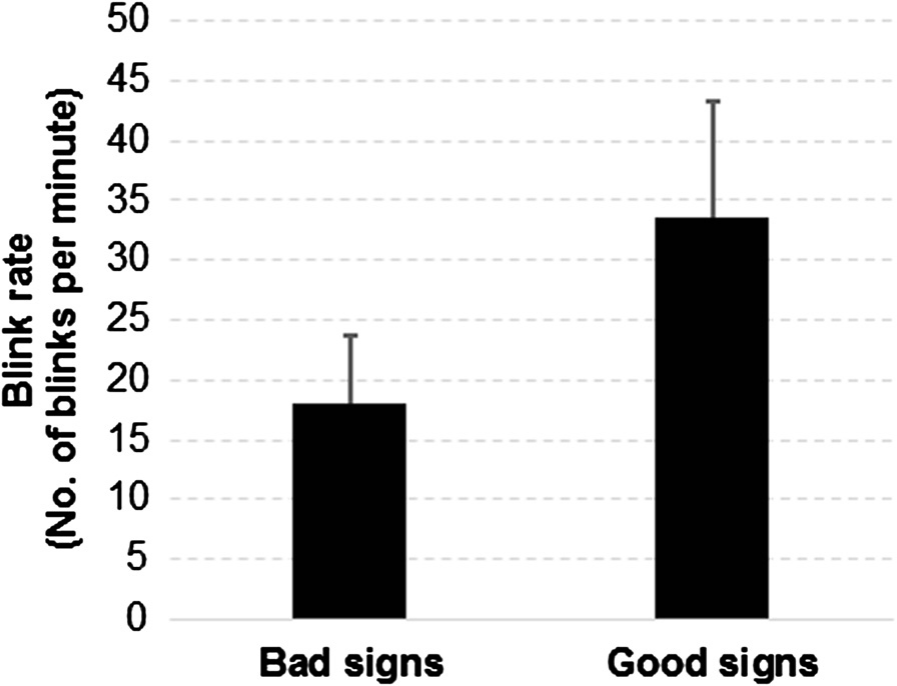
Differences in blink rates between bad and good signs

Differences in blink rates elicited by signs with different comprehensibility levels are shown in Fig. 4. It revealed that signs which were hard to comprehend (bad signs) elicited a significantly less number of blinks (18.0 blinks/min for bad signs vs. 33.6 blinks/min for good signs; *p* < 0.0001) compared to signs which were easy to comprehend (good signs).

#### Pupil diameter

**Fig. 5:**
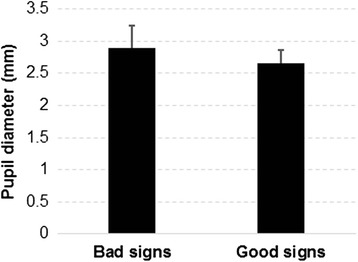
Differences in pupil diameters between bad and good signs

Significant differences in pupil diameters between the bad and good signs (*p* < 0.0001) were observed (Fig. 5). Bad signs elicited larger pupil diameters (an average of 2.9 mm), compared to the good signs (an average of 2.6 mm).

#### Time to first fixation

**Fig. 6:**
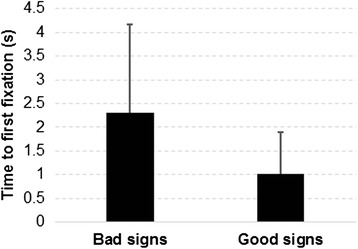
Differences in time to first fixation between bad and good signs

Figure 6 shows that participants spent a significantly (*p* < 0.0001) longer time to first fixation for the bad signs (an average of 2.3 s), compared to the good signs (an average of 1.0 s).

### EEG measures

**Fig. 7:**
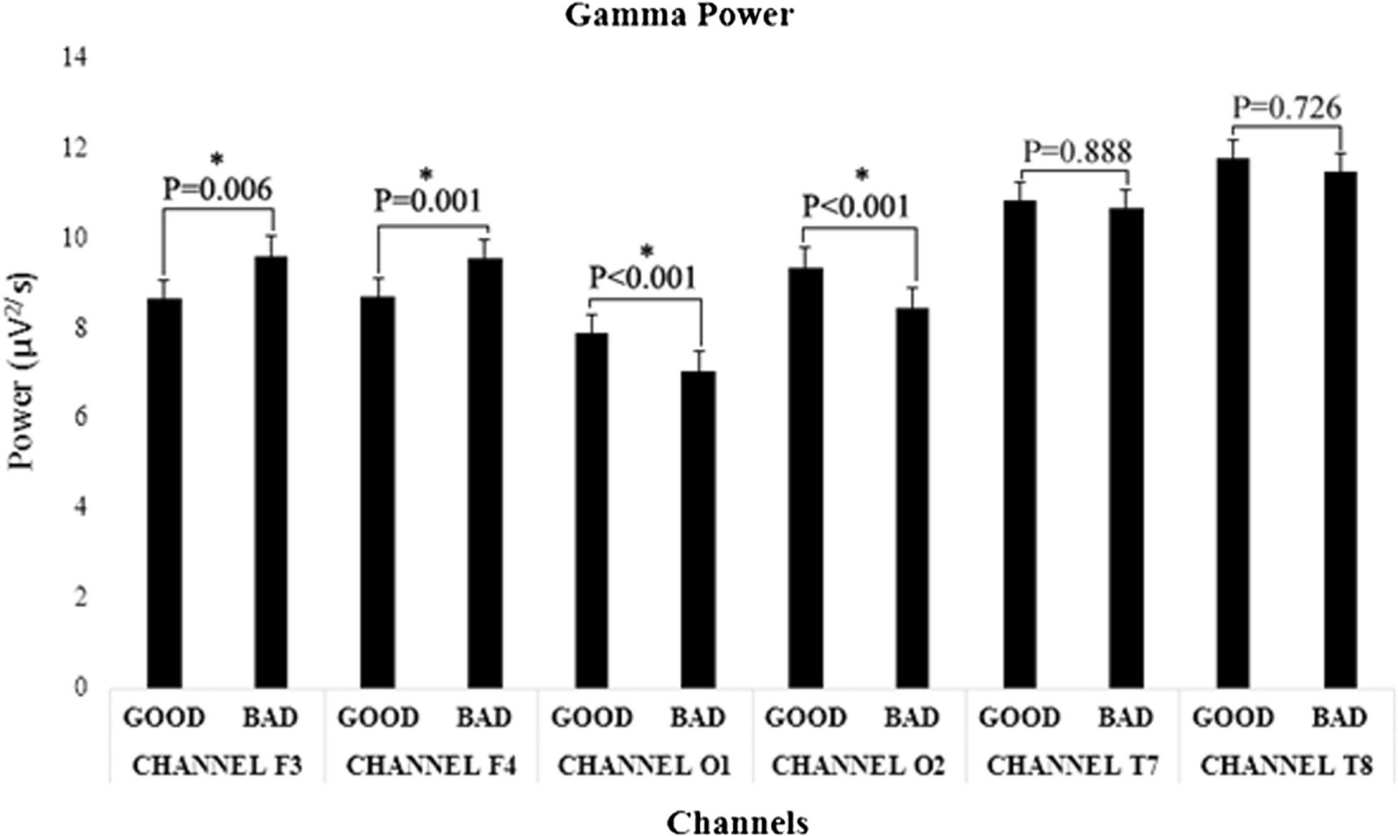
Gamma power differences between bad and good signs for each investigated channel (Significant differences between groups are marked as asterisks)

Figure 7 provides a typical example of experimental results on the gamma frequency band from power spectral analysis. Summarized results (Table 2) showed that even though there is no significant difference between the good and bad signs for delta band in all investigated channels, theta power is significantly and consistently higher for the good signs in both channel F3 (*p* = 0.002) and channel F4 (*p* < 0.001), which were located on the prefrontal cortex of human brain. With respect to the alpha band, spectral analysis results showed the good signs have significantly higher power than bad signs in all channels except channels T7 (*p* = 0.407) and T8 (*p* = 0.472). For the gamma band, the good signs have a significantly lower power than bad signs in channels F3 (*p* = 0.006) and F4 (*p* = 0.001) but a significantly higher power in channels O1 (*p* < 0.001) and O2 (*p* < 0.001).

**Table 2:** Effects of sign comprehensibility on investigated physiological measures from eye tracking and EEG data (*p* values less than 0.05 are shown in italics)

Physiological data		Measures	*p* value of two-group comparison (good signs vs. bad signs)
Eye tracking		Blink rate	*<0.001 (+)*
		Pupil diameter	*0.002 (−)*
		Time to first fixation	*<0.001 (+)*
EEG			
Prefrontal cortex (F3, F4)	Channel F3	Delta power	0.401
		Theta power	*0.002 (+)*
		Alpha power	*0.002 (+)*
		Gamma power	*0.006 (−)*
	Channel F4	Delta power	0.355
		Theta power	*<0.001 (+)*
		Alpha power	*<0.001 (+)*
		Gamma power	*0.001 (−)*
Visual cortex (O1, O2)	Channel O1	Delta power	0.996
		Theta power	0.102
		Alpha power	*<0.001 (+)*
		Gamma power	*<0.001 (+)*
	Channel O2	Delta power	0.233
		Theta power	0.189
		Alpha power	*<0.001 (+)*
		Gamma power	*<0.001 (+)*
Auditory cortex (T7, T8)	Channel T7	Delta power	0.906
		Theta power	0.445
		Alpha power	0.407
		Gamma power	0.888
	Channel T8	Delta power	0.646
		Theta power	0.470
		Alpha power	0.472
		Gamma power	0.726

Note: The plus sign (+) represents that the measure in good sign group is larger than the bad sign group; the minus sign (−) represents the opposite

**Fig. 8:**
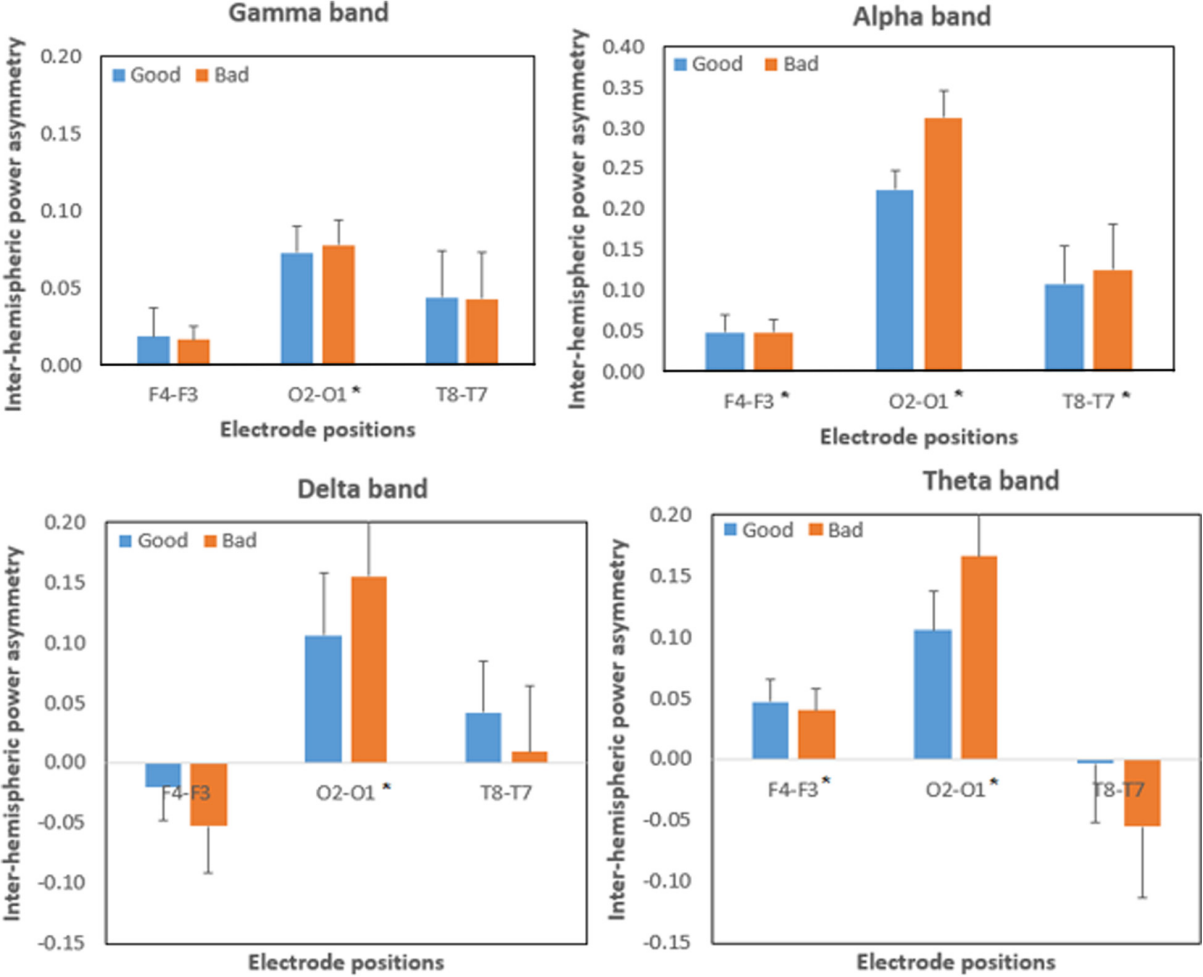
Mean (standard error) of inter-hemispheric power asymmetry indices [(*R* − *L*)/(*R* + *L*)] for good and bad signs with respect to frequency bands. The significant differences between the right and left hemispheres are marked as *asterisks*

Figure 8 summarizes the mean (standard error) of inter-hemispheric power asymmetry indices in the four frequency bands. Significant inter-hemispheric differences have been found with the O2–O1 site pairing for gamma, delta, theta, and alpha activities. Positive asymmetry indices for the O2–O1 site pairing indicate a greater right than left hemisphere power in the visual cortex during sign comprehension. Significant inter-hemispheric differences are limited to the alpha and theta bands for the F4–F3 site pairing and the alpha band for the T8–T7 site pairing. Additionally, regardless of the frequency bands, the power asymmetry indices for the O2–O1 site pairing were always much larger than the other two site pairings (F4–F3, T8–T7).

### Overall results of eye movements and brain oscillations to traffic symbols with different comprehensibility

Overall results for the investigated physiological measures are summarized in Table 2, which showed that 13 out of a total of 27 investigated physiological measures evoked significant changes when the participants were exposed to signs with different levels of comprehensibility (good and bad signs).

## Discussion

In the past few decades, a large body of safety sign research has examined how sign characteristics (such as color, shape, symbol, incongruent information) and receiver personal factors (such as age, gender, belief, perception of risk, stress) impact warning effectiveness [31]. These studies provide basic principles and guidelines for the design and implementation of more effective safety signs; however, the present study takes a further step by investigating the underlying human attention and cognitive processes that affect sign comprehension. This is the first study, to our knowledge, to investigate safety sign comprehensibility by utilizing physiological measures from eye tracking and spontaneous brain activity data. Real-time nonintrusive monitoring of human eye movements and brain oscillations during sign comprehension can provide us detailed and objective information on human attention and cognitive processes [17, 32].

**Fig. 9:**
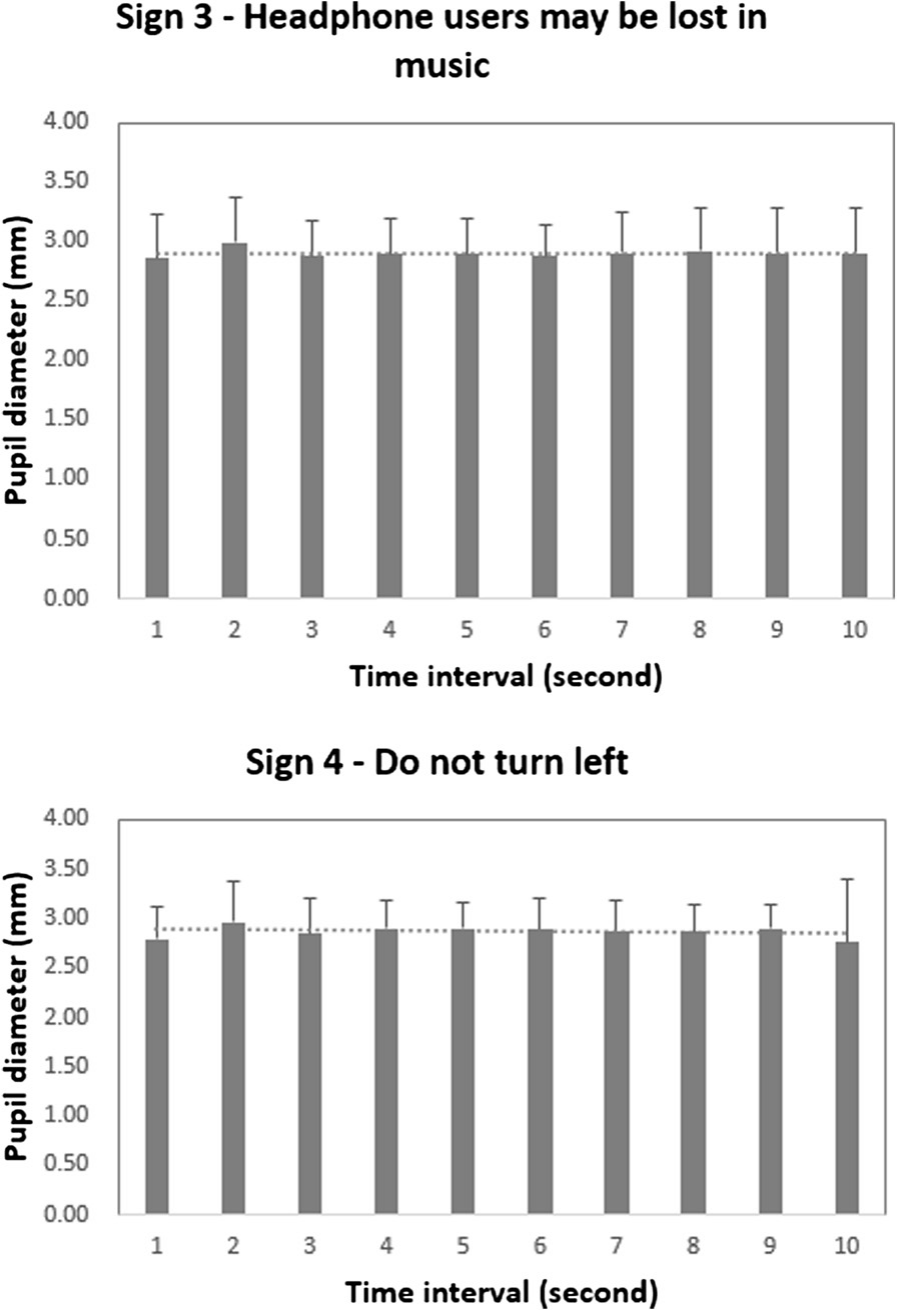
Pupil diameters at each 1-s time interval in the 10-s period for two representative signs (sign 3 and sign 4) in the different comprehensibility groups

In this study, we set a short period of time (10 s) and ask participants to look at each displayed sign and pay close attention to it, no matter if it is easy to comprehend or hard to comprehend. The participants should always stay focused on each sign for 10 s during the process of comprehension. Analysis on the temporal variation data of eye tracking showed that there is no significant change on eye movements, especially pupil diameter with the time (see Fig. 9 for typical examples) when a sign is displayed in the 10-s period. Therefore, the physiological differences should be largely affected by the different sign stimuli, and the confounding effects from the different actions of participants on comprehending two groups of signs (such as ceasing to pay attention to easy-to-comprehend signs during the later stage of the 10-s period but continuing to pay attention to hard-to-comprehend signs during whole 10-s period) should be minimal.

The results of this study demonstrated that the physiological measures of eye movements and the brain’s natural oscillations were able to reflect the changes from different comprehensibility levels of traffic symbols. The first and most prominent indicator found in this study was the blink rate, which was inversely related with comprehension difficulty. This result is consistent with previous studies. Holland and Tarlow [33] stated that blink occurs at the moment of cognitive change. For example, in some cases when participants were concentrating on finding an answer to a specific problem, they tend not to blink, and when they found the answer, blink occurs. Telford and Thompson [34] and Broadbent [35] reported that participants tend to suspend blinking while something interesting catches their attention and when information in memory is being operated on. In the case of safety sign comprehension, participants tend to perform less number of blinks while being exposed to a hard-to-comprehend sign, since they had to concentrate and focus their attention to the displayed sign in order to guess the actual meaning of the sign correctly. In addition, when compared to a normal blink rate of 24.36 blinks/min obtained from Cardona et al. [36], a decreased blink rate (18.0 blinks/min) was observed when participants were trying to comprehend a bad sign, suggesting that more cognitive load was required during this task. On the contrary, when participants were exposed to an easy-to-comprehend sign, it was expected that they would spend less cognitive load to guess the meaning of the sign, thus allowing faster blinks (33.6 blinks/min) when they set their attention loose.

In terms of pupil diameter, results showed that the increasing cognitive load was accompanied by an increasing pupil diameter within the time interval of 0.5–1 s following stimuli onset, resulting in larger pupil diameters for signs with low comprehensibility, in comparison with signs with high comprehensibility. This result is in line with previous studies which reported that pupil dilation is positively associated with increasing cognitive load [37–39]. Different comprehensibility levels of signs were also found to significantly affect time to first fixation. Results from our study suggested that participants had difficulties fixating their gaze on one part of the pictogram when exposed to signs with a low comprehensibility level, resulting in a longer time to first fixation. This result should be reasonable and can be supported by findings from previous studies [40, 41]. Salience could be another factor that affects time to first fixation [42, 43]. Pictograms used in the good signs probably contain a more prominent aspect that can be easily detected by the viewers compared with the pictograms used in the bad signs.

With regard to EEG signal, alpha, theta, delta, and gamma oscillations were examined in this study since they have been reported to govern cognitive processes [28]. Significant alpha, theta, and gamma power differences between good and bad signs were found in channels F3 and F4, which were parts of the prefrontal cortex of the brain. These results were reasonable, since the association between functions of the prefrontal cortex and various cognitive behaviors including orchestration of thoughts and actions and access to working memory had been acknowledged in many previous studies [44–49]. Miller and Cohen [48] reported that the prefrontal cortex plays a very important role in cognitive performance. They stated that representative areas in the prefrontal cortex can function as attentional templates by providing top-down signals to other parts of the brain which will guide the flow of activities needed to perform a cognitive task. More specifically, alpha power was observed to be higher for easy-to-comprehend signs in this study; this result was in line with a previous study by Klimesch [18] which suggested that an overall decrease in alpha power indicated an increasing cognitive load in general. According to Klimesch’s study in 1996 [50], alpha power was blocked or attenuated by attention and mental effort, which also explains the results obtained in the current study. Our study showed 9.9~13 % decreases of alpha power in both the prefrontal and visual lobes. Similar tendencies were also observed in a study with Alzheimer patients [50], where upper alpha power showed about 26 % decrease for tasks which require higher mental effort. Interestingly, about 9 % increase was also reported in the lower alpha power for tasks with higher mental effort. This finding is also noteworthy for future research, since dichotomy in the alpha frequency range could have led scientists to discover a more interesting nature of cognitive processes while encoding visual stimuli. Higher theta power was found for easy-to-comprehend signs since these signs can be encoded better compared to the bad signs [51, 52]. This result is also supported by a comprehensive study of brain oscillation from Klimesch [50], which suggested that theta oscillation is linked to an encoding process of new information. Lower gamma power in the prefrontal cortex (channels F3 and F4) was found for easy-to-comprehend signs; this could be explained by increasing gamma oscillations being possibly associated with the cognitive processing of attended stimuli [53]. In the current study, the gamma power in the prefrontal cortex showed 10.2~11.7 % increases, when participants were exposed with hard-to-comprehend signs. A similar pattern was also reported by Başar-Eroglu et al. [54], who investigated the visual perception of ambiguous patterns and reported 40~50 % increases in human frontal gamma activity, in comparison to spontaneous EEG recordings. The differences on the amount of changes in their study and our current study might be due to different cognitive tasks administered and the participants in the experiments. Many previous studies have showed that brain oscillations are correlated with multiple functions and are highly dependent on tasks, sensation, and individuals [28].

Aside from investigating channels in the prefrontal cortex, the present study also investigated the visual cortex of the human brain, represented by channels O1 and O2, since visual stimuli was utilized to capture the participant’s attention during the experiment. Higher alpha and gamma power was observed for easy-to-comprehend signs in both channels O1 and O2. Since overall decrease in alpha power indicated increasing demands of attention, alertness, and task load in general [18], the hard-to-comprehend signs could be associated with reduced alpha power. As to the gamma frequency band, higher gamma power was observed in channels O1 and O2 for the good signs since the occipital lobe is closely linked with visual saliency [55] and good signs likely evoke a higher visual saliency than the bad signs. However, it is also worthy to note that the increased gamma power observed in the visual cortex for the good signs in the current study might also be caused by other factors, such as stimulus properties or interindividual differences, which are often the causes of diverse findings in brain oscillation-related studies [50].

It was hypothesized in our study that differences between signs with different comprehensibility levels would exist only in specific brain regions related to visual stimuli processing and cognitive performance. Therefore, to prove this hypothesis, additional analysis was done for channels T7 and T8 located on the auditory cortex, which were supposed to be related with auditory stimuli processing [56]. The result showed no significant difference between the two groups of signs. This finding supported our hypothesis that spatial analysis focusing on brain regions related to attention, cognitive, and visual stimuli processing was an efficient approach to analyze brain oscillatory phenomena when comprehending the symbolic signs.

In coherence to cognitive processing of visual stimuli, laterality is one aspect that often draws interests. In this study, the brain oscillations in the delta, theta, alpha, and gamma tend to be more prominent in the right hemisphere of the visual cortex (O1 and O2). This result is relevant when compared to the previous studies, which mentioned that the right hemisphere of the human brain is specialized for information related to perception of a global shape, while the left hemisphere is specialized to process more detailed information, for example, when two or more modalities were involved [57]. Another study by Goldberg and Costa [58] also mentioned that the right hemisphere has a greater ability to process novel stimuli. It is noteworthy to mention that the tasks administered in the current study were stimulus-driven (bottom-up) and the obtained laterality pattern may differ when (1) instruction-driven tasks (top-down) are also incorporated into the experiment and (2) two or more modalities are incorporated into the experiment [59].

There are several limitations inherent in the current study. Firstly, the significant results obtained for several physiological measures investigated in this study were elicited by signs which had been proven to be very different in terms of comprehensibility level (easy to comprehend vs. hard to comprehend). Whether or not the significant measures found in this study can reflect human attention and cognitive processes elicited by signs whose comprehensibility levels are in between is definitely worthy of further investigation. Secondly, the stimuli used in the present study were traffic signs, and they were tested with young participants; whether the findings from this study can be generalizable to other types of safety signs and other populations should be studied in the future. Thirdly, a simple two-group comparison (easy-to-comprehend vs. hard-to-comprehend signs) is used in this study to provide some preliminary evidences that signs of different comprehensibility could induce some changes on simultaneously recorded physiological data. A further study with the addition of a control condition of “no sign” could be conducted to compare with sign conditions so that the effect of displaying sign stimuli on human physiological differences can also be examined. The last, but not least, advanced analysis techniques should be developed to link eye movement to the ongoing EEG for giving more information about what regions of the brain are activated while people engage in sign comprehension.

## Conclusions

This study investigated eye movements and brain oscillations to symbolic traffic signs with different comprehensibility. Results showed that the comprehensibility level of traffic signs significantly affects eye movements and EEG spectral power. The harder to comprehend the sign is, the slower the blink rate, the larger the pupil diameter, and the longer the time to first fixation. Noticeable differences on EEG spectral power between easy-to-comprehend and hard-to-comprehend signs can be observed in the prefrontal and visual cortexes of the human brain.

Taken together, these findings demonstrate the potential to integrate physiological measures from eye movements and brain oscillations with existing evaluation methods in assessing the comprehensibility of the symbolic safety signs. Real-time nonintrusive monitoring of human attention and the brain’s spontaneous electrical activity may enable researchers a deeper and advanced understanding the underlying cognitive processes that affect sign comprehension.

## Abbreviations

ANOVA: Analysis of variance
C-HIP: Communications–human information processing
EEG: Electroencephalogram
GS: Guessability score
