# Correction to: Eye movements and brain oscillations to symbolic safety signs with different comprehensibility

**DOI:** 10.1186/s40101-018-0191-9

**Published:** 2018-12-20

**Authors:** Yohana Siswandari, Shuping Xiong

**Affiliations:** 1 Department of Human and Systems Engineering, School of Design and Human Engineering, Ulsan National Institute of Science and Technology (UNIST), Ulsan, 689-798 South Korea; 2 Department of Industrial and Systems Engineering, College of Engineering, Korea Advanced Institute of Science and Technology (KAIST), Daejeon, 34141 South Korea

**Correction to: Journal of Physiological Anthropology (2015) 34:42**

**https://doi.org/10.1186/s40101-015-0081-3**

After the publication of the original article [1] it was highlighted that there was an omission regarding the online resources for the traffic signs in the section of “Experimental stimuli”. The single online source listed in the Fig. 2 should be removed and the exact detailed two online sources for the traffic signs should be cited in the section “Experimental stimuli”. This correction article shows the correct and incorrect version of this section and the caption of Fig. 2. The authors apologize for the inconvenience.

## Incorrect

### Experimental stimuli

Ten symbolic traffic signs (Fig. 2) were used as stimuli in this study. Among those signs, five widely used road signs (S1—do not turn right; S4—do not turn left; S7—U-turn is prohibited; S9—do not go straight; S10—turn right) were hypothesized to be easy to comprehend, and the other five new road signs in UK (S2—no vehicle carrying explosives; S3—headphone users may be lost in music; S5—tourist area; S6—caution texter; S8—risk of grounding) were hypothesized to be hard to comprehend.

Fig. 2 Ten symbolic traffic signs and their intended meanings (source: Know your traffic signs, Department of Transport, UK. www.gov.uk/)

## Correct

### Experimental stimuli

Ten symbolic traffic signs (Fig. 2) were used as stimuli in this study. Among those signs, five widely used road signs (S1—do not turn right; S4—do not turn left; S7—U-turn is prohibited; S9—do not go straight; S10—turn right) were hypothesized to be easy to comprehend, while the other five new road signs (S2—no vehicle carrying explosives; S3—headphone users may be lost in music; S5—tourist area; S6—caution texter; S8—risk of grounding) were hypothesized to be hard to comprehend. Among five new road signs, signs 2, 8 were retrieved from https://www.gov.uk/guidance/the-highway-code/ (The Highway Code: traffic signs, Department of Transport, UK) and signs 3, 5, 6 were retrieved from http://cabtradenews.org/2013/11/19/cab-drivers-call-for-new-road-signs-on-uk-roads/.

Fig. 2 Ten symbolic traffic signs and their intended meanings1
